# Head models of healthy and depressed adults for simulating the electric fields of non-invasive electric brain stimulation

**DOI:** 10.12688/f1000research.15125.2

**Published:** 2018-11-15

**Authors:** Nya Mehnwolo Boayue, Gábor Csifcsák, Oula Puonti, Axel Thielscher, Matthias Mittner

**Affiliations:** 1 Department of Psychology, Faculty of Health Sciences, University of Tromsø - The Arctic University of Norway, Tromsø, 9037, Norway; 2 Center for Magnetic Resonance, Department of Electrical Engineering, Technical University of Denmark, Kongens Lyngby, 2800, Denmark; 3 Danish Research Centre for Magnetic Resonance, Centre for Functional and Diagnostic Imaging and Research, Copenhagen University Hospital, Hvidovre, 2650, Denmark

**Keywords:** transcranial direct current stimulation (tDCS), transcranial magnetic stimulation (TMS), non-invasive brain stimulation (NIBS), major depressive disorder (MDD), Head models, computational modelling, magnetic resonance imaging (MRI), volume conduction model

## Abstract

During the past decade, it became clear that the electric field elicited by non-invasive brain stimulation (NIBS) techniques such as transcranial direct current stimulation (tDCS) and transcranial magnetic stimulation (TMS) are substantially influenced by variations in individual head and brain anatomy. In addition to structural variations in the healthy, several psychiatric disorders are characterized by anatomical alterations that are likely to further constrain the intracerebral effects of NIBS. Here, we present high-resolution realistic head models derived from structural magnetic resonance imaging data of 19 healthy adults and 19 patients diagnosed with major depressive disorder (MDD). By using a freely available software package for modelling the electric fields induced by different NIBS protocols, we show that our head models are well-suited for assessing inter-individual and between-group variability in the magnitude and focality of tDCS-induced electric fields for two protocols targeting the left dorsolateral prefrontal cortex.

## Introduction

Non-invasive brain stimulation (NIBS) techniques such as transcranial direct current stimulation (tDCS) and transcranial magnetic stimulation (TMS) have been used to investigate the relationship between activity in different cortical regions and cognitive processes ^1, 2^. A key advantage of NIBS is that it allows direct manipulation of neural excitability ^3^. Therefore when used carefully, it allows causal interpretation of how specific brain regions might be involved in mental phenomena such as perception ^4^, working memory ^5^, attention ^6^, decision-making ^7^ or emotional regulation ^8^. In addition, NIBS has been used as a clinical tool to obtain symptom reduction in several neurological and psychiatric conditions ^9– 12^. Importantly, the same stimulation protocol can result in different neural effects across individuals because the distribution of stimulation-induced electric fields (E-fields) in the brain is strongly contingent on anatomical variability ^13, 14^. This can manifest in strong variability in the effects of NIBS on cognitive performance in healthy individuals (e.g., working memory ^15, 16^) and on treatment outcomes in patients (e.g., depression ^17– 21^).

Given the heterogeneity in the efficacy of NIBS protocols in modulating behavior and clinical symptoms, there has been a move towards computational modelling of the spatial distribution of E-fields in the brain to understand how stimulation parameters such as electrode placement, electrode rotation or electrode type affects current flow in the neural tissue. After an initial phase during which simplistic spherical head models were used ^22^, the focus has shifted to very detailed, realistic head models created from individual structural magnetic resonance (MR) images using freely available tools (e.g. SimNIBS ^23^ or ROAST ^24^). Creating individual head models remains challenging, however, due to (1) the requirement of high-quality structural MR images for each study participant and (2) the need to manually improve the automatic segmentation of the MR images into the different tissue types (i.e., bone, cerebrospinal fluid, white and grey matter, air, etc). As a consequence, individual head models are rarely used in practice. Instead, researchers usually rely on E-fields induced in a reference individual which is generalized to all participants. This approach, of course, neglects the importance of individual brain anatomy which can have strong influences on E-field distribution ^13^.

To circumvent this issue, the New York (NY) Head model ^25^ was created from the ICBM152 (v6 template ^26, 27^ and v2009b template ^28^), which is a non-linear average of 152 individual MRIs and is extended to the full head and neck region by fusing it with an average of an additional 26 brains. Therefore, this head model represents an unbiased population average and should be a better representation of the individual participants than any randomly picked reference individual. However, calculation of the electric field on a single head model does not allow to quantify variation across individuals that can be substantial both in cortical regions directly below the electrodes and those that are farther away from the stimulation sites. Also, given that the NY Head was created using healthy individuals, possible systematic differences between patient groups may not be noticed.

To improve this situation, we present 38 head models created from MR images of 19 healthy participants and 19 patients with major depressive disorder (MDD). These head models were manually checked and edited to improve their accuracy in giving a faithful representation of cortical regions. Our head models can aid future research in at least three ways: First, calculating electric fields across a large sample allows to get a sense of the variability of the induced E-field due to anatomical differences. Second, our head models also enable comparison of the neural effects on NIBS protocols in healthy vs. depressed brains. Given the different protocols used for left dorsolateral prefrontal cortex (lDLPFC) targeting with NIBS ^29, 30^, computational modelling using these head models will enable the development of protocols for more selective lDLPFC stimulation in this disorder. Third, EEG source localization also relies on head models to calculate the spatial location of possible current sources in the brain. Usually, boundary element models (BEM) are used which have the limitation of less anatomical detail. Therefore, finite element models (FEM) derived from high resolution MR images have become more widespread because they are able to incorporate more tissue types, increasing the precision of EEG source localization. Our head models can be used for source localization using open-source software ^31^. Thus, our head models can help researchers to optimize NIBS protocols and EEG source localization methods, and to test them on a larger sample (including both healthy and patient data).

## Methods and results

### Participants

High-resolution head models were created from T1-weighted anatomical images that were collected in a separate functional magnetic resonance imaging (fMRI) study ^32^. The data was obtained from the OpenfMRI database (accession number: ds000171). Structural scans of 19 healthy adult participants with no history of depression or other psychiatric disorders (11 females; mean *±* SD age: 28.79 *±* 10.86; range: 18–59) and 19 individuals diagnosed with MDD and experiencing a depressive episode at the time of the scanning (11 females; mean *±* SD age: 33.52 *±* 13.35; range: 18–56) were used. Data of one control participant (’sub-control20’) was excluded due to technical problems with head model creation. Patients did not suffer from current or past manic episodes, current comorbid anxiety disorders or current alcohol dependence or abuse. At the time of data collection, all patients were unmedicated, but 6 received antidepressant pharmacotherapy in the past. For full details regarding demographic data, we refer to the supporting information of the original paper ^32^.

### Creation of head models

As the very first step, we inspected scans of all participants, and manually removed signals corresponding to the MRI marker placed on the forehead of each subject using FreeSurfer 5.3.0 ^33^. Automated tissue segmentation was performed in SPM12 ^34^ for skin, skull, eyeballs, CSF and major air cavities, and in FreeSurfer for gray and white matter. Subsequently, segmented images of each participant were visually inspected and manually corrected with FreeSurfer (done by investigator G.Cs., verified by O.P.). Manual corrections were primarily restricted to the skull-CSF boundary, but in some cases also involved the skin-skull interface. In addition, during manual corrections we verified that the segmentation of the cortical gray matter corresponded to the anatomical scans except for medial temporal lobe structures (i.e., the parahippocampal gyrus and the hippocampus proper). Moreover, the resulting masks were not corrected for inconsistencies relating to subcortical nuclei and thus, these head models are not suitable for estimating stimulation-related E-fields in structures such as the thalamus, basal ganglia, amygdala or the cerebellum. Additionally, the segmentation of the brainstem is not accurate because it arbitrarily assigned brain tissue to white and grey matter. Furthermore, because the original dataset was de-faced and did not include the neck/shoulder region, our head models do not include these regions. This has 2 implications: Firstly, this limits their usability regarding the simulation of tDCS montages with extracephalic return electrodes. Nevertheless, they can be used for all tES protocols using scalp electrodes and most TMS protocols. Secondly, an extended head model with field of view covering the entire head would further increase the predictive accuracy of the head models ^35^.

**Figure 1.**
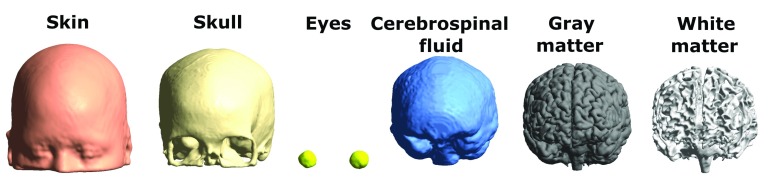
The six tissue compartments of the head models.

Head models were created with a custom version of SimNIBS 2.1 ^23^, a freely available software package for simulating the effects of NIBS techniques. The final head mesh of each participant consisted of a total number of approximately 3,200,000 tetrahedral elements, assigned to six tissue types ( Figure 1). The initial segmentation included more than 6 tissue compartments (e.g., separate tissue types for cerebellar gray and white matter; available in the m2m_sub-* folders) but they were later combined into one of 6 tissue types: skin, skull, CSF, GM, WM and eyeballs in the final head models for simulation purposes. In addition, air cavities were modeled by not adding tetrahedra to these locations, similar to the air surrounding the head. For comparability with other datasets, we also report measurements for head size: The distance between the nasion and inion (mean *±* SD: 19.2 cm *±*1.01 cm; range: 16.7 cm - 21.3 cm) and the distance between the right and left pre-auricular points (mean *±* SD: 14.8 cm *±*0.65 cm; range: 13.6 cm - 16.3 cm). The total volume of the brain was 1.22 dm ^3^ *±*0.11 dm ^3^ (range: 1.02 dm ^3^ - 1.49 dm ^3^). Individual measurements are found at our data repository ^36^

**Figure 2.**
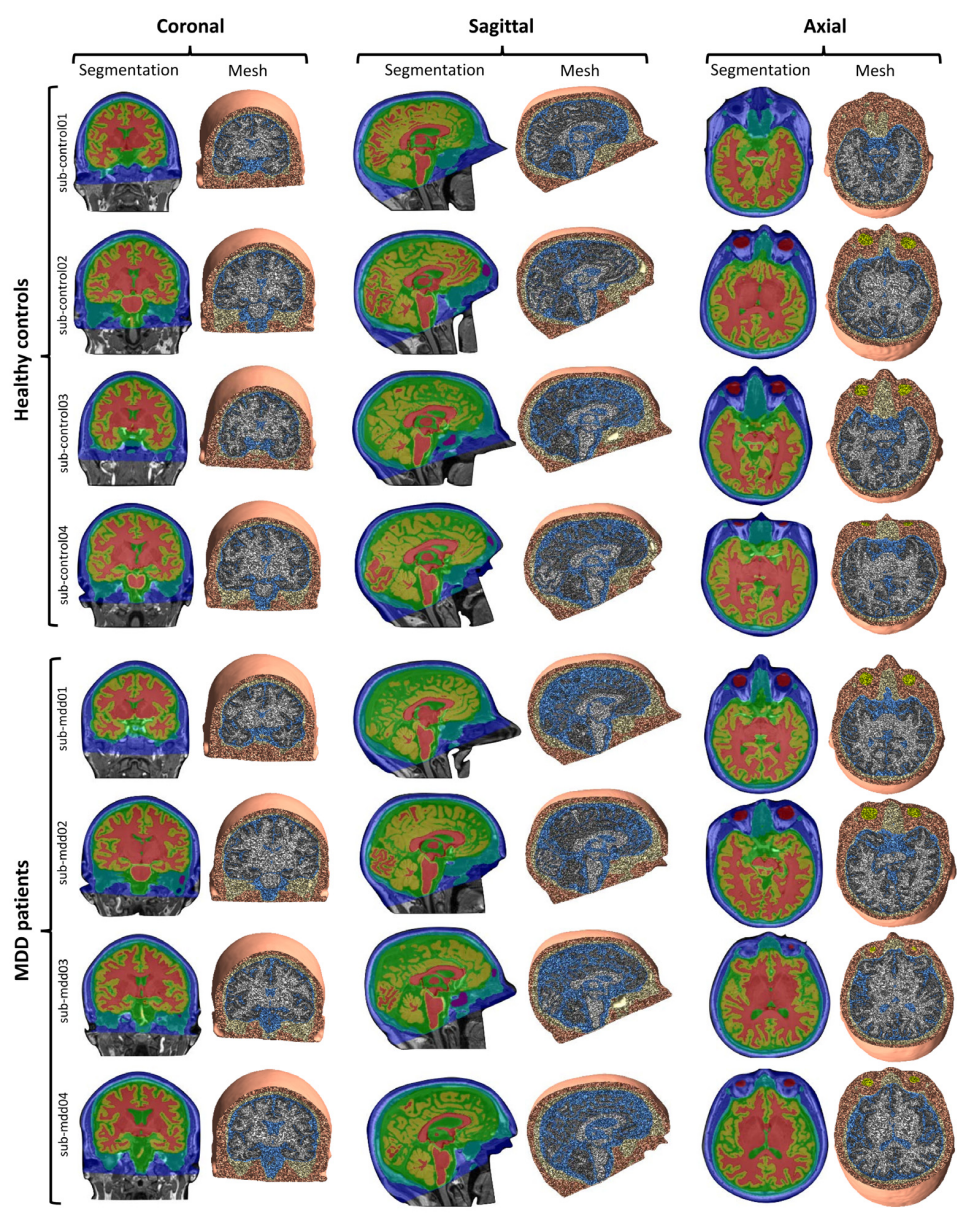
Cross-sections showing the correspondence between anatomical scans overlaid with results of the tissue segmentation (skin: dark blue; skull: turquoise; cerebrospinal fluid: green; gray matter: yellow; white matter: red; air cavities: purple, eyeballs: dark red) and the head models (meshes) for 4 individuals from both groups.

Tissue conductivities were set as follows: 0.465 S/m (skin), 0.01 S/m (skull), 0.5 S/m (eyeballs), 1.654 S/m (cerebrospinal fluid), 0.275 S/m (gray matter), 0.126 S/m (white matter). Although our head models do not account for white matter anisotropy, this property has been shown to primarily influence current density in deeper structures, leaving superficial gray matter relatively unaffected ^37^. The accuracy of tissue segmentation and the good correspondence between anatomical scans and the resulting head models for 8 individuals are shown in Figure 2.

## Dataset validation

Except for two manual steps (removal of MRI markers from the forehead, manual correction of tissue segmentations), the process of head model creation was automated using a custom version of SimNIBS 2.1 that employed FreeSurfer 5.3.0 for brain segmentation (as described in 38 and implemented in mri2mesh) and SPM12 for segmentation of the remaining tissues (similarly to 39 and implemented in headreco). This pipeline provides more accurate tissue segmentation relative to other protocols. It was a custom pipeline developed before the official release of SimNIBS 2.1. However, using headreco combined with the CAT12 toolbox (included with SimNIBS 2.1.1) for cortical reconstruction, the same accuracy can be achieved. We provide scripts compatible with SimNIBS 2.1.1 for automated simulation of tDCS-induced electric fields for all head models available for download at our data repository ^36^.

For validating the reliability of our head models, we compared the effects of two tDCS protocols targeting the lDLPFC (one conventional bipolar montage and one multi-electrode 4x1 setup) against the effects observed in the NY Head ^25^. The mesh for the NY Head (abaqus format; https://www.parralab.org/nyhead/) has been reformatted to be compatible with SimNIBS and is also available for download in our data repository ^36^.

**Figure 3.**
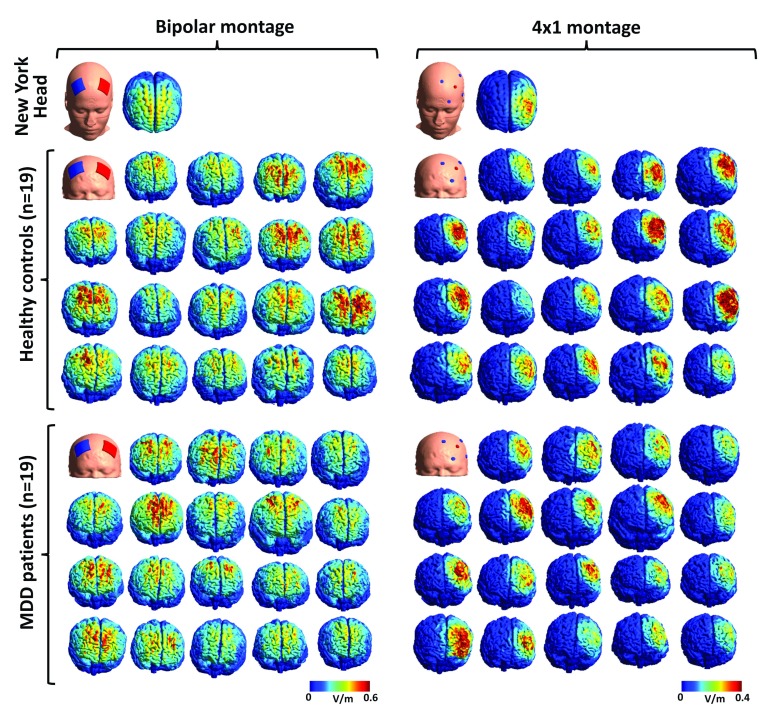
The spatial distribution of tDCS-induced E-fields in the NY Head and our 38 head models for the bipolar and the 4×1 montages. Please note the large degree of variability in E-field magnitude (both protocols) and in the lateralization of effects (bipolar protocol).

For each head model, tDCS electrodes of appropriate size (bipolar montage: 5 x 5 cm, circular connectors (diameter: 0.5 cm) at the middle of the electrode pads; 4x1 montage: diameter of 1.2 cm) and thickness (1 mm for all electrodes + a sponge pocket of 2.5 mm thickness for the bipolar montages and a gel layer of 2.5 mm thickness for the 4x1 montages) were placed at scalp locations corresponding to electrode positions of the International 10/10 system (bipolar montage: anode - F3, cathode - F4; 4x1 montage: anode: F3, cathodes: C3, FT7, Fp1, Fz). Stimulation intensity for the anode was set to 2 mA, with equal distribution of return currents for the 4 cathodes (0.5 mA for each) in the 4x1 protocol. Results of the simulations were visualized using Gmsh ^40^ ( Figure 3).

**Figure 4.**
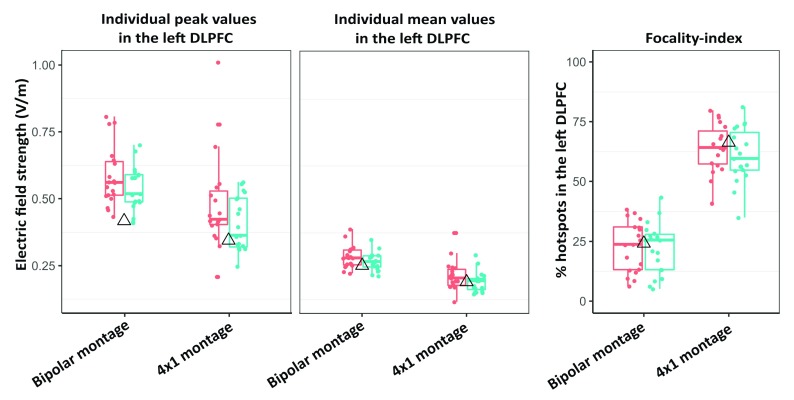
Variability of tDCS-induced E-field strengths across head models for the two montages. Both peak (left panel) and mean (middle panel) E-field magnitudes in the lDLPFC are stronger for the bipolar montage, whereas the 4×1 protocol yields more focal stimulation of the target region (right panel). Group means (red: healthy individuals, green: MDD patients) do not differ substantially, but large degree of inter-individual variability can be observed in both groups. The triangles show data for the New York Head. Horizontal lines within boxes represent median values, whereas lower and upper box hinges correspond to the first and third quartiles (25 ^th^ and 75 ^th^ percentiles). Lengths of upper/lower whiskers extend to the largest/smallest values that do not exceed 1.5* the inter-quartile range; dots represent individual data.

In our previous study ^14^, we reported stronger stimulation-induced E-fields in the lDLPFC for the bipolar montage used by Brunoni and colleagues (2013) ^17^ than for the 4x1 protocol, albeit the bipolar montage was also associated with reduced focality (i.e., more intensive stimulation of other cortical areas). Therefore, we extracted three measures for both tDCS protocols for our 38 head models: stimulation strength (the norm of the electric field vector, ’normfield’) in the lDLPFC was assessed by extracting individual mean (calculated across all nodes in this region) and maximum (peak) E-field values, whereas spatial focality of the stimulation was analyzed by calculating the focality-index (FI), with the target region as reference. FI was quantified as the proportion of highest-intensity nodes (nodes within the upper 1% percentile of all E-field values) in the lDLPFC relative to the whole cortex. Mean and peak E-field values corresponding to both tDCS montages were extracted by reconstructing the two-dimensional cortical surface (more precisely, the middle of the cortical sheet) of each individual along with the corresponding E-field cortical map in FreeSurfer, and an automated atlas-based parcellation of the frontal lobe ^41^ was applied to each individual brain to delineate the lDLPFC. As a result, we show that (1) both protocols induce strong E-fields in the DLPFC (with symmetrical effects for the bipolar montage and unilateral E-field distribution for the 4x1 protocol), (2) E-field magnitudes and distributions are similar for our head models and for the NY Head, (3) all E-field measures (peak and mean strength, FI) show great degree of variability, and (4) montage-specific effects are consistent with previous results reported in the literature regarding both the spatial distribution and the magnitude of E-fields ^35, 42– 45^ ( Figure 3, Figure 4). Accessing group differences between MDD and healthy subjects was not the primary aim of the current data note, however, analysis of the spatial distribution of tDCS-induced E-fields in the bilateral DLPFC and medial prefrontal cortex showed subtle group differences between the healthy and MDD groups. For detailed discussion of these results and that of Figure 4 we refer the reader to our accompanying paper ^14^.

## Data availability

*Open Science Framework (OSF)*: Dataset 1. Head models of healthy and depressed adults for simulating the effects of non-invasive brain stimulation, http://doi.org/10.17605/OSF.IO/EXBD5 ^36^

License: CC0 1.0 Universal

At our data repository ^36^, the following data are available for download for all subjects (healthy adults: ’sub-control’, patients: ’sub-mdd’): T1-weighted anatomical scans registered to FreeSurfer conform space (’nii.gz’ files), the corresponding segmentation masks for the 7 (6 tissue types of the final meshes + a mask for major air cavities) tissue types (’nii.gz’ files), the final head models (’msh’ files), the files containing electrode coordinates (International 10/10 system) for all participants (’txt’ files can be used for performing new script-based simulations, whereas the ’geo’ files can be used to plot electrode positions directly onto the mesh files in Gmsh ^40^), and files organized in 2 folders (’fs_*.tar.gz’ and ’m2m_*.tar.gz’) that enable creating simulation outputs in average FreeSurfer space (’fsaverage’). We also included a README file with a detailed description of the data and scripts.

### Usage notes

Our head models are compatible with SimNIBS 2.1.1 ( http://simnibs.de/) for simulating the effects of tDCS and TMS protocols. This software package has an easy-to-use graphical user interface (GUI) for setting all stimulation parameters (scalp location, intensity, etc.) for both NIBS techniques. By using our custom-written script ^36^, it is also possible to run tDCS simulations for any given montage for all participants at once. The script will also output data registered to an average surface (’fsaverage’) which allows creating group averaged data, as we have shown previously ^14^. In addition, researchers have the opportunity to extract E-field components that are either radial (normal) or tangential relative to the cortical surface, and have been associated with different cellular effects ^46^. At our data repository ^36^ we also provide the manually corrected segmentations for the different tissue types for those who would like to create high-quality meshes of their own using open-source software such as iso2mesh ^47^. Finally, our meshes can be used for improving the anatomic precision of EEG source localization, using open-source tools ^31^.
