# Supplementary material for: Head models of healthy and depressed adults for simulating the electric fields of non-invasive electric brain stimulation
Source: F1000Res. 2018 Nov 15;7:704. Originally published 2018 Jun 6. [Version 2] doi: 10.12688/f1000research.15125.2 (PMC6241565; doi:10.12688/f1000research.15125.2)
Supplement: Peer review history

# Peer review: Referee response for version 2

**DOI:** 10.5256/f1000research.18435.r40676

**Published:** 2018-11-15

**Authors:** Yu Huang

**Affiliations:** 1 City College of New York, New York City, NY, USA

The authors have addressed all my concerns.

I have read this submission. I believe that I have an appropriate level of expertise to confirm that it is of an acceptable scientific standard.

# Peer review: Referee response for version 1

**DOI:** 10.5256/f1000research.16478.r37428

**Published:** 2018-09-17

**Authors:** Yu Huang

**Affiliations:** 1 City College of New York, New York City, NY, USA

This is a modeling effort trying to predict how electric field distributes under trascranial electric stimulation for up to 19 normal and 19 pathological subjects. It is well written and a good contribution to the literature. The authors are advised to address the following issues before indexing:

 1) Only transcranial electric stimulation (TES) is done in this work. There is no simulation on magnetic stimulation (TMS). So the title of this manuscript should be “non-invasive *electric* brain stimulation”

 2) The authors only discussed there are inter-individual variabilities in E-field distributions, but did not say anything about how the E-field differs (or is similar) between the healthy subjects and depression subjects, and why.

 3) As shown in Figure 1, the head models are all cut off without the lower part of the head. This will give significant difference in the E-field distributions compared to a head model that covers the entire head (Huang and Liu, et al, 2017 ^1^). Please discuss this as one limitation of this work.

 4) There are results in the Section Methods. So the section name should be “Methods and Results”.

 5) It is unclear that how the lDLPFC region was separated. You only mentioned “...applying automated atlas-based parcellation of the frontal lobe to delineate the lDLPFC region in each brain”. Please briefly describe how this was done. Was some atlas registered to each individual brain to extract the region?

 6) You said SimNIBS 2.1 was used, but did not mention which function was used for the segmentation. In SimNIBS 2.1, “headreco” calls SPM12 to segment all the head tissues, and “mri2mesh” uses FreeSurfer to segment gray and white matter, and FSL to segment non-brain tissues. You said “...SimNIBS 2.1 that employed FreeSurfer 5.3.0 for brain segmentation (as described in 29) and SPM12 for

 segmentation of the remaining tissues (similarly to 30).” This is not clear to me. Did you combine “headreco” and “mri2mesh”? Also if you used SPM12, there should be a tissue type “air cavities”, but from Figure 1, this “air cavities” is not there.

 7) The thickness of electrodes in your models “1 mm for all electrodes + 2.5 mm sponge pocket/gel

 layer for the bipolar and 4×1 montages, respectively” is not clear. Please clarify this sentence.

 8) Minor:

- In “Introduction” section, the first 3 sentences in the first paragraph are general statements but lack references. Please add.
- In “Introduction” section, 2 ^nd^ paragraph, what do you mean by “rotation or type”?

I have read this submission. I believe that I have an appropriate level of expertise to confirm that it is of an acceptable scientific standard, however I have significant reservations, as outlined above.

## References

[1] HuangYLiuALafonBFriedmanDDayanMWangXBiksonMDoyleWDevinskyOParraL: Measurements and models of electric fields in the in vivo human brain during transcranial electric stimulation. *eLife*.2017;6: 10.7554/eLife.18834 10.7554/eLife.18834 PMC537018928169833

# Response

**Published:** 2018-10-26

**Authors:** Nya Boayue

**Affiliations:** University of Tromsø, Norway

We are grateful to Dr. Huang for reviewing our manuscript. Your comments have helped give the revised version of our manuscript more clarity. We have addressed all the points raised in the new version of the MS. We have also updated the scripts at our data repository such that they are compatible with the currently released SimNIBS (version 2.1.1).

1. The title of the new manuscript has been changed based on your suggestion.
2. We chose to keep the focus here on the data since this is a data note. However, we have added the following to the **Dataset validation** section. “Accessing group differences between MDD and healthy subjects was not the primary aim of the current data note, however, analysis of the spatial distribution of tDCS-induced E-fields in the bilateral DLPFC and medial prefrontal cortex showed subtle group differences between the healthy and MDD groups. For detailed discussion of these results and that of Figure 4 we refer the reader to our accompanying paper https://www.sciencedirect.com/science/article/pii/S0165032717324746.”
3. Many thanks for this very important point and pointing us to the article. This has now been addressed in our manuscript as one of the limitations of using our head models in the ***creation of head models*** *section* . “... an extended head model with field of view covering the entire head would further increase the predictive accuracy of the head models https://elifesciences.org/articles/35178”
4. The Methods section has been renamed to “Methods and Results”.
5. Through personal communication with the authors of https://onlinelibrary.wiley.com/doi/full/10.1002/hbm.22309, we received a script which was used for the automatic parcellation of the frontal lobe. We have now clarified this aspect: “an automated atlas-based parcellation of the frontal lobe https://onlinelibrary.wiley.com/doi/full/10.1002/hbm.22309 was applied to each individual brain to delineate the lDLPFC.”
6. This is a good point. The reason for the lack of clarity is that we used a custom version of the software which, at the time, resulted in better results than using the latest released version. In the meantime, a newer SimNIBS version has been released. We have added the following detail to the MS to avoid any misunderstandings. “… the process of head model creation was automated using a custom version of SimNIBS 2.1 that employed FreeSurfer 5.3.0 for brain segmentation (as described in https://onlinelibrary.wiley.com/doi/full/10.1002/hbm.21479 and implemented in mri2mesh) and SPM12 for segmentation of the remaining tissues (similarly to https://www.sciencedirect.com/science/article/pii/S1053811918301800 and implemented in headreco). This pipeline provides more accurate tissue segmentation relative to other protocols. It was a custom pipeline developed before the official release of SimNIBS 2.1. However, using headreco combined with the CAT12 toolbox (included with SimNIBS 2.1.1 ) for cortical reconstruction, the same accuracy can be achieved.”As for the lack of “air cavities” in the Figure 1, we have added the following clarification in the ***creation of head models*** *section “…* *air cavities were modeled by not adding tetrahedra to these locations, similar to the air surrounding the head* *.* *”*
7. This sentence has now been clarified. It reads “ 1 mm for all electrodes + a sponge pocket of 2.5 mm thickness for the bipolar montages and a gel layer of 2.5 mm thickness for the 4x1 montages.”
8. *We have now added the required references. *
9. *We meant “* *electrode rotation or electrode type* *”* ***.*** *this has been corrected in the MS.*

# Peer review: Referee response for version 1

**DOI:** 10.5256/f1000research.16478.r34789

**Published:** 2018-06-18

**Authors:** Ilkka Laakso

**Affiliations:** 1 Department of Electrical Engineering and Automation, Aalto University, Espoo, Finland

Non-invasive brain stimulation techniques are based on generating, either magnetically or electrically, electric fields that can alter brain neuronal activity. However, the generated electric fields can vary greatly depending on the individual anatomy of the scalp, skull and brain. This data note presents a collection of 38 individual head models (both healthy and patients) that can be used for characterisation of variability in the electric fields.

 Head models were constructed from T1-weighted MRI using freely available software: FreeSurfer, SPM12 and SimNIBS. Manual verification and corrections were applied when necessary. The approach is state of art.

 The datasets are provided in the Gmsh format, which can be used directly in modelling software or converted to multiple other formats using open-source software. Furthermore, raw data, including FreeSurfer subject data, are included, allowing further processing and adaptation of the data.

 Minor comments:

 1. In the text and Figures 1 and 2, it is written that the head models are segmented to six tissue types. Actually, it seems that there are more than six tissue compartments, as cerebellar GM and cerebellar WM are separate from the cerebral GM and WM. Also, at least some air cavities are segmented (missing from the caption of figure 2).

 2. Brainstem is segmented as cerebellar white/grey matter. It may be helpful to list this as a limitation, for instance, in "Creation of head models".

 3. In the abstract, sentence "... effects of non-invasive brain stimulation ...", and in "Dataset validation", sentence "The scripts used for automated simulation of tDCS effects for all head models ...".

 Electric fields are not "effects".

I have read this submission. I believe that I have an appropriate level of expertise to confirm that it is of an acceptable scientific standard.

# Response

**Published:** 2018-10-26

**Authors:** Nya Boayue

**Affiliations:** University of Tromsø, Norway

We would like to thank Dr. Laakso for reviewing our manuscript and for the helpful comments. These comments have been addressed in the new version of the manuscript. We have also updated the scripts in our data repository such that they are compatible with the currently released SimNIBS (version 2.1.1).

  

1. *Thanks for pointing out this issue. We have now added air cavities to the caption of Figure 2. Concerning the discrepancy between the segmentations and the final head models, we have now added the following clarification to the* ***creation of head models*** *section “* The initial segmentation included more than 6 tissue compartments (e.g., separate tissue types for cerebellar gray and white matter; available in the m2m_sub-* folders) but they were later combined into one of 6 tissue types: skin, skull, CSF, GM, GM and eyeballs in the final head models for simulation purposes. In addition, air cavities were modeled by not adding tetrahedra to these locations, similar to the air surrounding the head *.* *”*
2. This is an important point. We have now added the following sentence to the ***creation of head models*** *section* “Additionally, the segmentation of the brainstem is not accurate because it arbitrarily assigned brain tissue to white and grey matter.  
3. We concur with this point. The abstract sentence now reads “… the electric field elicited by non-invasive brain stimulation ...” The " **Dataset validation**" sentence which was changed a bit now reads “ We provide scripts compatible with SimNIBS 2.1.1 for automated simulation of tDCS-induced electric fields for all head models ...”

                                                                                                                                
